# Prevalence of Fabry Disease in Patients With Cryptogenic Strokes: A Systematic Review

**DOI:** 10.7759/cureus.19358

**Published:** 2021-11-08

**Authors:** Juan Fernando Ortiz, Jashank Parwani, Paul W Millhouse, Ahmed Eissa-Garcés, Gashaw Hassen, Victor D Cuenca, Ivan Mateo Alzamora, Mahika Khurana, Domenica Herrera-Bucheli, Abbas Altamimi, Adam Atoot, Wilson Cueva

**Affiliations:** 1 Neurology, Larkin Community Hospital, Miami, USA; 2 Neurology, Lokmanya Tilak Municipal Medical College, Mumbai, IND; 3 General Practice, Drexel University College of Medicine, Philadelphia, USA; 4 Neurology, Universidad San Francisco de Quito, Quito, ECU; 5 Medicine and Surgery, University of Parma, Parma, ITA; 6 Progressive Care Unit, Mercy Medical Center, Baltimore, USA; 7 Medicine, Universidad San Francisco de Quito, Quito, ECU; 8 Public Health, University of California Berkeley, Berkeley, USA; 9 Medicine, Universidad Internacional del Ecuador, Quito, ECU; 10 Emergency Medicine, Amiri Hospital, Kuwait City, KWT; 11 Internal Medicine, Palisades Medical Center, North Bergen, USA

**Keywords:** alpha-galactosidase, cryptogenic stroke, fabry disease, stroke, ischemic stroke

## Abstract

Fabry disease (FD) is an X-linked disorder involving multiple organs. Stroke is a serious and frequent complication of FD. Cryptogenic stroke is a common presentation of FD, especially in the young population. The etiology of cryptogenic stroke is highly variable and difficult to assess, frequently leaving patients without a primary diagnosis. We conducted a systematic review to investigate the pooled prevalence of FD among patients with cryptogenic stroke, or patients with FD in whom a stroke was the presenting condition. English-language studies involving humans published in the last 20 years were included in this systematic review. FD was more common in male patients and tended to present at an earlier age. The frequency of hemorrhagic and ischemic strokes in this population was similar to that in the general population. There was a high rate of stroke recurrence in the study sample, even among patients undergoing enzyme replacement therapy. We conclude that screening for FD in patients with cryptogenic stroke is low yield and not cost-effective. However, it may be worthwhile to screen for FD among patients with recurrent strokes.

## Introduction and background

Fabry disease (FD) is an X-linked lysosomal storage disorder caused by an inborn error in the glycosphingolipid metabolic pathway, with deficient activity of the lysosomal enzyme alpha-galactosidase A that results in lysosomal accumulation of globotriaosylceramide (Gb3) in multiple organs [1]. Gb3 mainly accumulates in the endothelial, cardiac, renal, and dorsal root ganglion neuronal cells [2]. The prevalence of FD is approximately 1 in 117,000 individuals [1]. The majority of cases are reported in Caucasians, and most patients who present with symptoms are male [2]. There are different types of FD mutations on the *alpha-galactosidase* (*GAL*) gene which causes the disease, including point mutations (83.4%), missense mutations (50%), nonsense mutations (29.2%), frameshift mutations (16.6%), and splicing (4.2%) [1].

Suspicion of FD can come through family history, clinical manifestations, and lab abnormalities, with the diagnosis confirmed by biochemical and molecular genetic testing [3]. Neuroradiologic evaluation is done using T2-weighted MRI, fluid-attenuated inversion recovery (FLAIR), or via evidence of pulvinar hyperintensity on T1-weighted images. These lesions are present in one-third of patients and are very suggestive of the disease when the diagnosis is uncertain [4].

There are two types of FD, namely, classical and late-onset FD. The classical form occurs in hemizygous males or heterozygous females [5]. Classical FD initiates at a young age with the main symptoms including acroparesthesias, gastrointestinal disturbances, angiokeratomas, impaired sweating, and heat and cold intolerance [5]. Complications include hypertrophic cardiomyopathy, renal failure, and stroke [5]. Female heterozygotes are also symptomatic, especially when they have skewed X chromosome inactivation [5]. Patients with late-onset FD usually present in the third to the seventh decade of life. Most patients do not display the classical symptoms as just one organ may be affected. The level of enzyme activity ranges from 2% to 30% of the normal, and an accumulation of Gb3 on blood vessels may not be evident [6-8].

There is a dearth of large-scale studies on FD. Although many observational studies have described the prevalence of stroke and FD, most included small cohorts. To reduce bias and determine a more accurate record, a pooled analysis of observational studies is needed. In this review, we aim to determine the prevalence of FD in patients with cryptogenic stroke or first-time stroke. We further aim to consolidate the knowledge of cryptogenic stroke and FD, explore the pathophysiology of ischemic stroke in FD, investigate the use of enzyme replacement therapy (ERT) as a preventive measure for stroke recurrence, and explore the influence of gender among these patients.

## Review

Methodology

Protocol

This systematic review was conducted following the Meta-Analysis of Observational Studies in Epidemiology (MOOSE) reporting guidelines [9].

Eligibility Criteria and Study Selection

Only observational studies conducted among human subjects in the last 25 years and written in the English language with the full report available were included in this review. We excluded case reports, systematic reviews, literature reviews, and meta-analyses that did not fulfill the study criteria. We included studies reporting on patients aged 18 to 60. Additionally, any articles that were not deemed relevant to the study parameters were excluded.

After screening the results, only articles with at least one of the following characteristics were included: patients with cryptogenic ischemic stroke or stroke presenting as the first symptom in whom measurement of either plasma levels of Gb3 with *α-GAL-A* gene sequencing, or α-GAL-A enzyme levels, was done. Patients with a pathogenic mutation were compared to those with a mutation of unknown pathogenicity. Our primary aim was to calculate the prevalence of FD in cryptogenic stroke. The secondary aim was to describe the characteristics of patients with cryptogenic stroke who were diagnosed with FD. We investigated the mutation type, stroke type, and prognosis of each of these patients.

Database and Search Strategy

For this systematic review, we used PubMed as a database. The search was done between July 25, 2020, and July 18, 2021. We used the following combination of search terms: “Fabry disease”[Title/Abstract] AND “stroke”[Title/Abstract]) OR (“cryptogenic stroke”[Title/Abstract] AND “Fabry disease”[Title/Abstract]. In addition to PubMed, Embase and Scopus were searched, but it did not result in any additional studies that fit the criteria.

Data Extraction

The following information was collected: author, year of publication, country of publication, study type, methodology, outcomes, and prevalence of FD in stroke patients. For all patients who presented with stroke and had a diagnosis of FD, the following additional information was extracted: type of mutation, Gb3 levels or plasma α-GAL-A levels, type of stroke, and prognosis.

Bias Analysis

To assess and minimize bias the risk of bias in non-randomized studies (ROBINS-I) tool for observational studies was used [10].

Results

**Figure 1:**
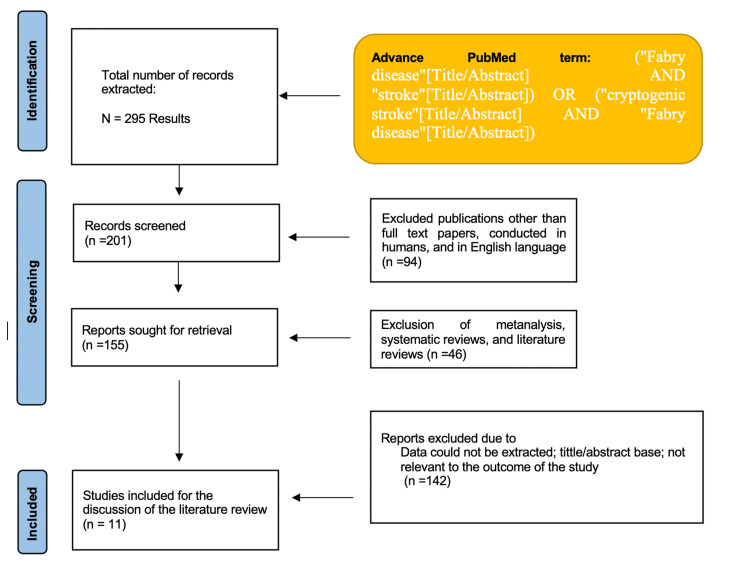
PRISMA flowchart of the systematic review. PRISMA: Preferred Reporting Items for Systematic reviews and Meta-Analyses

Figure 1 shows a flowchart with the results of this systematic review.

Study Characteristics and Outcomes

A total of 11 studies from 10 different countries were included. Probable and definitive diagnosis was assessed in the final prevalence among the observational studies. Table 1 shows the characteristics and outcomes of the cohorts investigated in this systematic review [1,2,11-19].

**Table 1:** Study type, methods, and outcomes of the studies. FD: Fabry disease; α-GAL-A: alpha-galactosidase-A; Gb3: globotriaosylceramide; TIA: transient ischemic attack; NIHSS: National Institutes of Health Stroke Scale; mRS: modified Rankin scale; GVUS: genetic variants of unknown significance; CT: computed tomography; MRI: magnetic resonance imaging

Author, year of publication, country	Study type	Methodology	Outcome
Duboc et al., 2011, Canada [2]	Single-center cohort study	Data were gathered from patients aged 16-55 years with cryptogenic stroke in a single center. α-GAL-A gene sequencing and plasma Gb3 levels were measured	Seven patients had a positive screening for FD (five women and two men) carrying mutations of undetermined pathogenicity. One patient had a mutation of unknown pathogenicity and another patient had confirmed pathogenic mutation and elevated levels of Gb3. Prevalence: 2/101 (1.9%) patients with cryptogenic stroke had FD
Lanthier et al., 2017, Canada [11]	Prospective cohort study	Enrolled patients aged 18-55 with either ischemic stroke or TIA. α-GAL-A gene was measured. NIHSS was measured acutely and mRS at seven days and six months	There were 8/397 patients who had a positive screening for FD. Six patients had multiple neutral genetic variants of the gene. One patient presented with a single disease-neutral variant (p.D313Y). Another patient presented with GVUS (p.R118C). The prevalence of FD was 1 in 395 (0.3%)
Gündoğdu et al., 2017, Turkey [1]	Prospective cohort study	484 patients aged 18-55 were enrolled from the city of Sakarya with acute ischemic stroke. Ischemic stroke was confirmed by CT and MRI. α-GAL-A was measured. Low enzyme levels of <1.2 μmol/L/hour were indicative of FD. DNA sequencing for GAL missense mutation for those with low enzyme activity was also done	484 patients with ischemic stroke were enrolled. 67 patients fulfilled the criteria of the study. 13 were lost to follow-up; hence, 54 patients were finally included in the study. Three (two males and one female) patients had low levels of α-GAL-A enzyme activity. The female had no gene mutation despite having low levels of the enzyme. The two male patients presented pathogenic mutation of FD (missense mutation). The prevalence of FD in this cohort was 2/67 (3.7%)
Malavera et al., 2020, Australia [12]	Prospective multicenter cohort study	The study included 326 patients aged 18-55 with cryptogenic stroke during 2014-2015. The levels of α-GAL A enzyme activity followed by gene analysis and lyso-Gb3 levels was conducted on 58/326 patients who consented for participation in the study	Out of 326 patients who met the inclusion criteria, 58 provided consent. However, six failed the initial screening blood draw and two withdrew consent. There were 50 participants who underwent enzyme/genetic analysis. 47% (n = 23) were females and 53% (n = 27) were males. Although 7/27 males showed low enzyme levels, genetic analysis did not reveal FD. Among the 23 females, only one had a pathologic FD on genetic analysis. Overall, the prevalence of FD was 1.72% in this cohort 1/58 (1.72%)
Rolfs et al., 2005, Germany [13]	Multicenter prospective cohort study	Between February 2001 and December 2004, 721 German adults aged 18-55 in 27 clinical departments in Germany suffering from cryptogenic stroke were screened for FD. The plasma α-GAL-A activity in men was measured, followed by sequencing of the entire gene in those with low enzyme activity. By contrast, the entire α-GAL gene was genetically screened for mutations in women even if enzyme activity was normal	32 patients had reduced activity of the enzyme (22 men 10 women). Out of them, 28 patients (21 men and 7 women) had a pathogenic mutation of the gene. Prevalence in males was 4.9%, and prevalence in females was 2.4%. Strokes were more common in the vertebrobasilar artery (42.9%). The total prevalence in this cohort was 28/721 (3.8%)
Kinoshita et al., 2012, Japan [14]	Multicenter prospective cohort study	From April 2015 to December 2016, 516 young patients (<60 years) with either ischemic or hemorrhagic stroke were enrolled in the study. α-GAL enzyme activity and Gb3 levels were measured followed by α-GAL-A gene sequencing	110 patients had cryptogenic strokes. Five patients (one woman and four men) had low α-GAL-A enzyme activity). Two patients had a mutation of unknown pathogenicity. No pathogenic mutation was found despite five patients having low levels of α-GAL-A activity. The prevalence of FD was 0/110 (0%) in this cohort
Alhazaa et al., 2020, Saudi Arabia [15]	Single-center cohort study	51 patients aged 18-55 with cryptogenic stroke were included in the study. α-GAL-A enzyme was measured, and genetic testing was performed in patients with abnormal results	500 patients were screened in this study, but only 51 were included. One case had borderline levels of the enzyme, but the genetic test revealed no genetic mutation of FD. Prevalence in this cohort was 0/51 (0%)
Brouns et al., 2007, Belgium [16].	Multicenter cohort Study	During January 2000 and December 2004, 103 patients with cryptogenic stroke aged 16-60 were screened for FD. Measurement of the α-GAL-A enzyme was followed by genetic testing in males with abnormal results	103 patients were included in the cohort. Three patients (two women and one man) had abnormal levels of the α-GAL-A enzyme. The same three patients had normal genetic testing. No patient was identified with FD. The prevalence in this cohort was 0%
Sarikaya et al., 2012, Switzerland [17]	Cohort study	Between January 2006 and October 2009, 150 patients were recruited, with a mean age of 43. Enzyme activity was measured in all patients. Genetic analysis was done in all females and males with low enzyme levels	150 patients met the inclusion criteria of the study. Nine patients had low enzymes levels (six men and three women). A nonpathogenic mutation was found on genetic analysis of the nine patients with low enzyme activity. However pathogenic mutation was not found. Hence, the prevalence in this cohort was 0%
Reinsin et al., 2018, Argentina [18]	Cohort study	Young patients in Argentina with TIA, ischemic stroke, or hemorrhagic stroke between 2011 and 2015 were enrolled. α-GAL-A levels were measured in all patients. Males with low levels of the enzyme and all females underwent genetic sequencing of the gene	311 patients were enrolled in the study. Ischemic events occurred in 89%, and 11% had a hemorrhagic event. Only one patient had the pathogenic mutation of FD. Another patient had a mutation of unclear significance. Both were missense mutations. Prevalence in this cohort was 2/311 (0.64%)
Wozniak et al., 2010, United States [19]	Cohort study.	550 men aged 15-49 years with their first ischemic stroke were enrolled in the study. The levels of α-GAL-A were measured in all patients. Patients with lower levels of the enzyme underwent genetic analysis	154 out of 550 patients with stroke had a cryptogenic stroke. 110 patients had low levels of the enzyme. Genetic testing on these patients showed a pathogenic mutation in one patient and a mutation of undetermined significance in another. Hence, the prevalence was 1/154 (0.65%)

We found 36 patients with cryptogenic stroke, or stroke as a presenting condition, in the 11 studies involving 1,986 patients in various cohort studies. The total prevalence of FD among these studies was 1.81%.

Stroke Characteristics of Patients With Fabry Disease

Table 2 shows the age and sex, mutation, Gb3 levels and enzyme activity, infarct type, outcome (second stroke), long-term outcome, and treatment of patients included in the studies [1,2,11-13,19].

**Table 2:** Characteristics of the patients with FD and stroke. Gb3: globotriaosylceramide; GVUS: genetic variants of unknown significance; ERT: enzyme replacement therapy; TIA: transient ischemic attack; mRS: modified Rankin scale; MCA: middle cerebral artery; PCA: posterior cerebral artery; α-GAL-A: alpha-galactosidase-A; MRI: magnetic resonance imaging; WMHs: white matter hyperintensities; CKD: chronic kidney disease; GI: gastrointestinal; FD: Fabry disease

Author, year	Age and sex	Mutation	Gb3 levels and enzyme activity	Infarct	Outcome (second stroke)	Outcome	Treatment
Dubuc et al., 2012 [2]	40 M	Unknown pathogenicity (GVUS)	Mildly elevated; Gb3 after the second stroke was normal	Nonlacunar vertebro basilar	42 months later, he had an infarct in the carotid artery territory	Bad functional	No ERT, antiplatelet drugs
	41 F	Pathogenic	Not described	Lacunar ischemic stroke	Two TIAs and two recurrent nonlunar ischemic strokes in the subsequent 63 months of follow-up	Memory impairment	Aspirin, ERT after stroke
Lanthier et al., 2017 [11]	55 M	GVUS (p.R118C)	Normal (Gb3)	Lacunar (left basal ganglia and subcortical infarctions). No leukoaraiosis	He had no further cerebrovascular events at seven months	mRS had improved from 3 at baseline to 1 at six months.	No information available
	39 F	Single disease-neutral	Normal (Gb3)	Lacunar (acute left thalamic lacune)	No recurrent cerebrovascular events on aspirin and atorvastatin prophylaxis	No information available	No information available
Rolfs et al., 2005 [13]	38.4 M	α-GAL gene	Men: α-GAL-A high or low + Gb3	MCA infarction: 28.6% (6/21). PCA infarction: 14.1% (3/21). Vertebrobasilar artery: 9/21 (42.9%). Hemorrhage: 5/7 (71.4%)	10 cases of multiple cerebrovascular events	Hemiparesis, ataxia, dysarthria, pathologic nystagmus	No information regarding ERT
	40.3 F		Women: α-GAL-A high or normal + Gb3	MCA infarction: 42.9% (3/7). PCA infarction: 14.3% (1/7). Vertebrobasilar artery: 4/7 (57.1%). Hemorrhage: 1/7 (14.3%). Vertebrobasilar infarction: 42.9%	Two cases of multiple cerebrovascular events	Information not available	Information not available
Malavera et al., 2020 [12]	49 F	Pathogenic	Normal levels of Gb 3	Intraparenchymal hemorrhage	No recurrent cerebrovascular events	Information not available	Secondary prevention with antiplatelets and statins
Gündoğdu et al., 2017 [1]	24 M	Pathogenic	Low α-GAL-A activity (0.1 μmol/L/hour)	Right basal ganglia infarction. MRI revealed WMHs in both hemispheres and lacunar infarctions in the thalamus, brain stem, and parietal lobe	No further cerebrovascular events at six months	Cardiomyopathy, CKD, neuropathic pain, hypohidrosis, and GI disease	ERT
	43 M	Pathogenic	Low α-GAL-A activity (0.1 μmol/L/hour)	Lacunary infarction in the pons (same place as the previous stroke)	Second lacunar stroke	Remained with left-sided paralysis.	ERT
Wozniak et al., 2010 [19]	Information not available	Pathogenic	Low α-GAL-A activity (0.1 μmol/L/hour)	Information not available	Information not available	Information not available	Information not available
	Information not available	Undetermined pathogenicity	Low α-GAL-A activity (0.1 μmol/L/hour)	Information not available	Information not available	Information not available	Information not available

Among the stroke patients, there were 32 ischemic infarcts (86.48%) and five hemorrhagic infarcts (13.51%). The number of patients with re-infarctions was 16 (43.24%). The mean age of males was 37.24 and for females was 41.11 years. In general, Gb3 levels were normal or mildly elevated in patients for whom the value was available. Measurement of α-GAL-A activity was a more sensitive marker than Gb3 level. Among the cohorts, there was heterogeneity in the way the different studies described mutations. We included patients with pathogenic mutations, mutations of unknown pathogenicity, and neutral mutations with related pathogenicity.

Study Limitations

Table 3 shows the bias of the studies included in this systematic review [1,2,11-16,19].

**Table 3:** Bias analysis using ROBINS-I tool. ROBINS-I: risk of bias in non-randomized studies of interventions

Author, year	Confounding	Selection of participants	Classification	Deviations	Missing data	Measurements	Selection of reported results
Gündoğdu et al., 2017 [1]	Medium risk	Moderate risk	Low risk	Low risk	Moderate risk	Low risk	Low risk
Dubuc et al., 2012 [2]	Low risk	High risk	Low risk	Low risk	Low risk	Low risk	Low risk
Lanthier et al., 2017 [11]	Low risk	Moderate risk	Low risk	Low risk	Low risk	Moderate risk	Low risk
Malavera et al., 2020 [12]	Low risk	Moderate risk	Low risk	Low risk	Low risk	Moderate risk	Low risk
Rolfs et al., 2005 [13]	Low risk	Low risk	Moderate risk	Low risk	Low risk	Moderate risk	Low risk
Kinoshita et al., 2018 [14]	Low risk	Moderate risk	Low risk	Low risk	Low risk	Low risk	Low risk
Alhazzaa et al., 2020 [15]	Low risk	High risk	Low risk	Low risk	Moderate risk	Low risk	Moderate risk
Brouns et al., 2007 [16]	Low risk	Moderate risk	Low risk	Low risk	Low risk	Moderate risk	Low risk
Wozniak et al., 2010 [19]	Low risk	High risk	Low risk	Low risk	Moderate risk	Low risk	Moderate risk

In five studies, the authors found that the sample was too small to reach statistical power [1,11,14-16]. In the study by Kinoshita et al., genetic analysis was not conducted in each patient, which could lead to a measurement bias [14]. Lanthier et al. stated that they could have missed some FD cases as all patients did not undergo genetic analysis [11]. In the study by Alhazzaa et al., the inclusion criteria were somewhat restrictive and more than half of the cohort suffered regular vascular risk factors such as diabetes, hypertension, and/or dyslipidemia, which could have contributed to the stroke. Further, in the study by Brouns et al., genetic testing was not performed among women which could introduce bias as the enzyme levels in female FD patients are often normal (false negatives) [16]. Finally, this study did not include cases of lacunar infarcts which further limited their numbers [15].

Discussion

Pathophysiology of Stroke in Patients With Fabry Disease

Most studies agree that the accumulation of glycosphingolipids in vascular endothelium causes disturbances in vasoreactivity and autoregulation, increasing the likelihood of stroke. Other theories have also been suggested in the literature. According to Sawada et al., vasospasm may play an important role in the pathophysiological mechanism of stroke in FD patients. This is supported by the high incidence of spastic coronary angina seen in FD [20]. Hilz et al. extensively studied stroke mechanisms and concluded that reduced cerebral blood flow velocities and impaired autoregulation are key factors [21]. A review by Rombach et al. suggested that smooth muscle cells are primarily involved in the vasculopathy of FD and that, in the early stages of Fabry vasculopathy, angiotensin II production becomes upregulated [22]. Further, other mechanisms such as increased reactive oxygen species production, enhanced nitric oxide levels, and the presence of a prothrombotic state have also been identified to contribute to the development of stroke [23]. Lastly, cardiac arrythmias, hypertrophic cardiomyopathy and myocardial infarction may lead to stroke in patients with FD [14].

Stroke in Patients with Fabry Disease and Prevalence of Fabry Disease in all Types of Strokes

FD affects both the peripheral nervous system and the central nervous system (CNS). The CNS symptoms in FD include psychiatric manifestations, headaches, and strokes [20]. In this systematic review, we only included information on cryptogenic strokes or patients with stroke as the first sign of presentation; therefore, all patients in our sample pool were, by definition, late-onset FD patients. Stroke is approximately 12 times more common in patients with FD than in the general population [1]. Approximately 25% of FD patients develop transient ischemic attack and stroke at a mean age of 40 (PM1) [24]. From the FD database (PM2), 138 out of 2,446 patients developed stroke [24]. The median age of first-time stroke was 39 years in males and 45.7 in females. Ischemic stroke was seen in 86.8% of the patients and hemorrhagic stroke in 13.9% of the patients, which is very similar to the general population [24]. In the same study, 70.4% of the strokes were lacunar [24]. Five of 52 females (8.6%) and 43 of 86 males (50%) who experienced stroke did not have the diagnosis of FD [25]. In comparison to our review, the rates of ischemic and hemorrhagic strokes were similar, and the age of presentation and the ratio of males to females were also similar. Table 4 lists the studies that included all types of strokes [25-27].

**Table 4:** Studies of all type of strokes and FD. FD: Fabry disease; CVS: cardiovascular stroke; α-GAL-A: alpha-galactosidase-A

Author, year, country	Number of cases	Prevalence of FD
Lee et al., 2019, Taiwan [25]	1,000 stroke patients: 661 ischemic strokes and 339 hemorrhagic strokes.	Two male patients with ischemic stroke were found to have a pathogenic mutation of FD. Overall prevalence was 0.2 for all strokes and 0.3 for ischemic strokes
Rolfs et al., 2013, Europe [26]	5,023 patients with CVS from 15 different countries were enrolled in this study. Ischemic stroke: 3,396, hemorrhagic stroke: 271, transient ischemic attack: 1,071	Definitive FD was found in 0.5% of the sample (N = 27). Probable FD was found in 0.3% of patients (N = 18)
Song et al., (2016), China [27]	357 patients underwent tests for α-GAL-A activity. 293 out of 357 patients with cerebral infarction and 64 out of 357 patients with transient ischemic stroke were screened for mutations in the enzyme	No patients with FD were found (prevalence: 0%)

Prevalence in studies that included all types of strokes ranged from 0% to 0.5% compared to our pooled analysis (0-3.88%), suggesting that the prevalence of FD is higher with cryptogenic strokes than other stroke types. The study by Rolfs et al. reported the highest prevalence among all the studies [26]. Most studies included only patients who developed a stroke for the first time. However, the study by Rolfs et al. also included patients with recurrent strokes, suggesting that the prevalence of FD among patients with recurrent stroke is higher compared to the general population.

Screening Fabry Disease in Patients With Ischemic Stroke

Early recognition of FD in patients with cryptogenic stroke is critical to prevent recurrent strokes [11]. However, most studies agree that generalized screening for FD among these patients is expensive and has low yield due to the low disease prevalence [2]. However, patients with recurrent strokes in some cohorts had higher rates of FD, suggesting that screening could be beneficial in this population [11,26].

Females tend to have normal levels of α-GAL-A enzyme activity due to the random inactivation of chromosome X, making gene sequencing more essential among females [12]. Measuring the levels of Gb3 has shown to be a more effective screening tool than α-GAL-A enzyme activity among females. However, measuring the enzyme activity is a more sensitive test in males [14]. Gündoğdu et al. suggested that screening of FD in countries with a high prevalence of genetic disease could be beneficial [1]. Moreover, recognizing neutral mutations is vital to ensure that patients do not go through unnecessary investigations and treatment [11]. Alhazzaa et al. also argued that screening for FD in patients with cryptogenic stroke is not cost-effective [15].

Management and Role of Enzyme Replacement Therapy in the Treatment of Fabry Disease

ERT has been shown to improve kidney function, reduce cardiomyopathy, and improve circulation by increasing NO levels. However, the prevention of stroke with ERT has not been demonstrated [20]. There is no consensus regarding when to initiate ERT in FD. Some experts suggest that it should be started among men as soon as the diagnosis is made and among women if clinical manifestations are present. The initial consensus suggests that ERT does not prevent stroke. However, a pooled analysis including seven cohort studies and two randomized controlled trials indicated that ERT has a beneficial effect in preventing stroke and stroke recurrence in patients with genetically, enzymatically, and/or biopsy-proven FD [28]. In one report, stroke recurrence rates were 0.082 in the ERT group versus 0.160 in the control group, with the difference being statistically significant [28]. The effects of ERT occurred gradually, but prevented strokes in the long term [29]. Sheng et al. suggested that women should consider initiating ERT at the time of FD diagnosis because stroke often occurs before the diagnosis is made [28].

Rolfs et al. argued that patients should be on a more effective antiplatelet medication such as clopidogrel or a combination of dipyridamole and aspirin [13]. Only two studies in our systematic review described patients who received ERT after stroke. In these studies, three patients received ERT but two developed stroke recurrence. We recommend further research to help determine whether ERT prevents recurrent strokes in patients with cryptogenic stroke due to FD.

## Conclusions

Although the pathogenesis of stroke in FD remains unclear, various mechanisms such as vasospasm, reduced cerebral blood flow velocity, abnormal autoregulation and upregulation of angiotensin II, and accumulation of glycosphingolipids in vascular endothelium have been proposed.

The prevalence of cryptogenic stroke in FD is highly variable among studies. However, compared to single strokes, FD was seen more commonly in patients with recurrent strokes. Hence, it may be useful to perform FD screening in such cases. Screening should be done by determining α-GAL-A enzyme activity in males and plasma Gb3 levels in females, followed by *α-GAL-A* gene sequencing for confirmation.

Finally, we conclude that ERT may decrease the recurrence of stroke in FD patients in the long term. Despite the initial consensus that ERT does not prevent stroke, the difference was statistically significant in recent systematic reviews.
